# Promoter Hypermethylation of CHODL Contributes to Carcinogenesis and Indicates Poor Survival in Patients with Early-stage Colorectal Cancer

**DOI:** 10.7150/jca.38815

**Published:** 2020-02-28

**Authors:** Xinyue Zhang, Kaiming Wu, Yuhua Huang, Lixia Xu, Xiaoxing Li, Ning Zhang

**Affiliations:** 1 Department Of Gastroenterology, The First Affiliated Hospital, Sun Yat-sen University, Guangzhou, China, 510080; 2 Precision Medicine Institute, The First Affiliated Hospital, Sun Yat-sen University, Guangzhou, China, 510080; 3 Gastrointestinal Surgery Center, The First Affiliated Hospital, Sun Yat-sen University, Guangzhou, China, 510080; 4 Department Of Pathology, Sun Yat-sen University Cancer Center, Guangzhou, China, 510080

**Keywords:** CHODL, methylation, prognosis, carcinogenesis, colorectal cancer

## Abstract

**Aims:** Aberrant hypermethylation of CpG islands is an important hallmark of colorectal cancer (CRC). We previously utilized methyl-DNA immunoprecipitation assays to identify a novel methylated gene, chondrolectin (CHODL), preferentially methylated in human CRC. In this study, we examined the epigenetic inactivation, biological effects and prognostic significance of CHODL in CRC.

**Main methods:** The methylation status of CHODL in CRC was evaluated by bisulfite genomic sequencing (BGS). The functions of CHODL in CRC were determined by proliferation, apoptosis, cell migration and invasion assays. The impact and underlying mechanisms of CHODL in CRC were characterized by western blot and RNA-Seq analyses. The association between CHODL and CRC clinical features was examined using The Cancer Genome Atlas (TCGA) database and immunohistochemical staining.

**Key findings:** CHODL was downregulated in 10 CRC cell lines and CRC tissues, and promoter hypermethylation contributed to its inactivation. Ectopic expression of CHODL inhibited colony formation, suppressed cell viability, induced apoptosis, and restrained cell migration and invasion *in vitro* and *in vivo*. Furthermore, high CHODL expression in CRC was a predictor of improved survival, though CHODL hypermethylation was a poor prognostic factor for CRC patients, especially those with early-stage CRC.

**Significance:** CHODL promoter hypermethylation silences CHODL expression in CRC, and CHODL suppresses CRC tumorigenesis. CHODL methylation and expression levels can be used as potential markers to evaluate the prognosis of CRC patients.

## Introduction

Colorectal cancer (CRC) is the third most common cancer in males and the second most common cancer in females, and CRC is the fourth leading cause of cancer-related death worldwide, with approximately 1.4 million new cases per year 1. The 5-year survival rate of CRC is less than 65%, and the morbidity rate has increased rapidly over the last 30 years in many countries 2. The mechanisms underlying the carcinogenesis of CRC are still unclear, attracting increased interest from researchers. The development of CRC is a complicated, multistage process that can arise from an accumulation of genetic and epigenetic changes, such as chromosome instability (CIN), microsatellite instability (MSI), the C-phosphate-G (CpG) island methylator phenotype (CIMP) and global DNA hypermethylation 3,4. The CIMP, characterized by extensive hypermethylation of multiple CpG islands in the promoter regions of genes, was introduced by Toyata and colleagues in 1999 5. Currently, the CIMP is considered one of the major tumorigenic pathways in CRC for the transcriptional silencing of tumor suppressor genes 6-7. The identification of novel tumor suppressor genes regulated by the CIMP may provide insight into the tumor suppressive pathways in CRC carcinogenesis and reveal potential biomarkers for tumor diagnosis.

By utilizing methyl-DNA immunoprecipitation assays, we previously identified a novel methylated gene, chondrolectin (CHODL), which is preferentially methylated in human CRC 8. Located at chromosome 21q21, CHODL belongs to the C-type lectin-like domain superfamily, and lectins play an important role in the cytoplasm, cytoplasmic membrane, and extracellular space 9. Through reverse transcription polymerase chain reaction (PCR) and immunohistochemistry (IHC) image analysis, Weng L et al. demonstrated that CHODL is primarily expressed in the vascular muscle of the testis, smooth muscle of the prostate stroma, heart muscle, skeletal muscle, and crypts of the small intestine 10. Aberrant CHODL expression was found in spinal muscular atrophy mouse models 11. However, the function of CHODL in tumors has not yet been reported. Masuda et al. indicated that CHODL was overexpressed in non-small-cell lung cancer (NSCLC), which contributed to a poor prognosis in patients, and the exogenous transfection of CHODL into a lung cancer cell line enhanced cell growth and invasion 12. Our recent research on CHODL in hepatocellular carcinoma (HCC) showed that the expression of CHODL was substantially decreased in HCC samples and cell lines 13. Nonetheless, the functional role of CHODL in CRC progression remains unclear.

In this study, we demonstrate that promoter hypermethylation of the CHODL gene contributes to its silencing in CRC. Further functional experiments revealed that CHODL suppresses CRC cell proliferation, induces apoptosis, and inhibits cell migration and invasion *in vitro* and *in vivo*. Moreover, through analysis of The Cancer Genome Atlas (TCGA) database and IHC staining, we found that CHODL mRNA expression and methylation levels are related to the survival of CRC patients. High CHODL expression in CRC was a predictor of improved survival, and the hypermethylation of CHODL serves as a poor prognostic factor for CRC patients, especially those with early-stage CRC. Our results demonstrate the epigenetic mechanism and tumor suppressive function of CHODL in CRC.

## Results

### CHODL is downregulated in primary colorectal tumors

**Figure 1:**
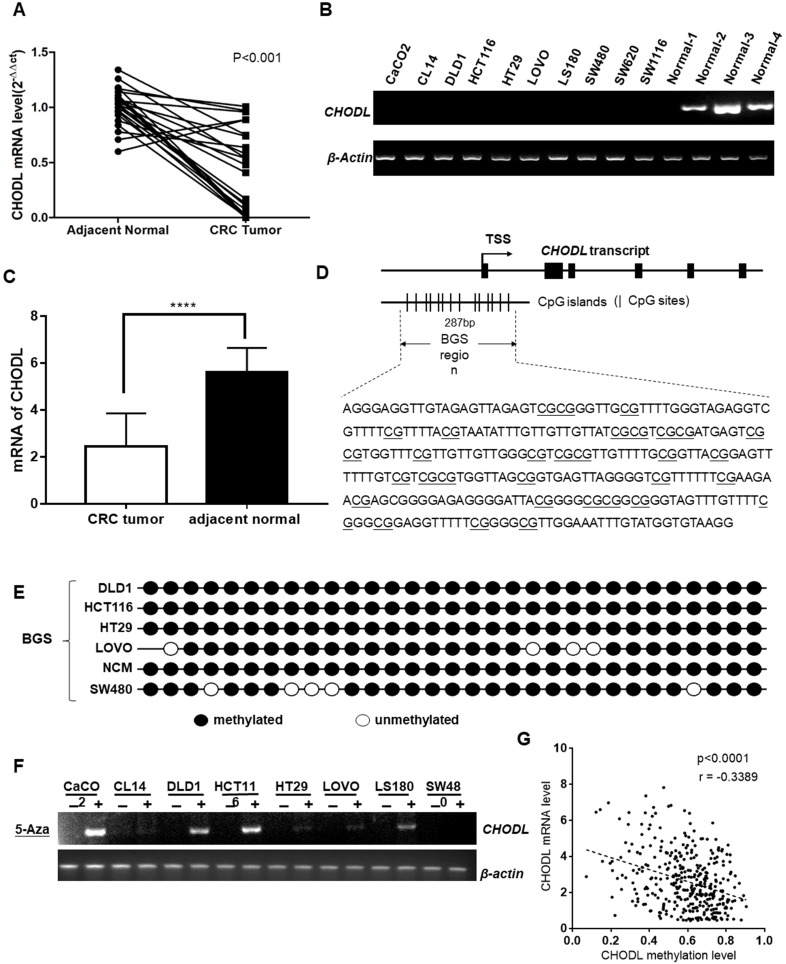
** CHODL silencing was linked to DNA methylation.** (A) CHODL mRNA levels in 24 paired primary CRC samples were measured by real-time PCR with β-actin as a control. (B) CHODL mRNA levels in 11 CRC cell lines and 4 normal tissue samples were examined by RT-PCR with β-actin as a control. (C) Differences in the mRNA levels of CHODL in TCGA between CRC tissues and adjacent normal tissues. (D) The BGS region relative to the transcription start site of genomic CHODL. (E) The methylation status was further confirmed by BGS. (F)CHODL mRNA expression was partially restored after treatment with the demethylation reagent 5-Aza-dC in 9 CRC cell lines. (G) Correlation between the level of CHODL promoter methylation and its expression (data from Xena).

First, we examined the mRNA expression of CHODL using real-time PCR in 24 paired CRC tumor tissues and their adjacent nontumor samples. The results showed that CHODL was significantly downregulated in 92% (22/24) of the CRC tumor tissues compared with its expression in the adjacent nontumor tissues (p<0.0001; Figure 1A). Then, we corroborated this by analyzing TCGA data from 597 CRC cases, and the results were consistent with *our in vitro* data (i.e., CHODL was downregulated in CRC tissues compared with adjacent normal tissues) (Figure 1C). CHODL downregulation was also observed in all 10 CRC cell lines compared with normal colon tissues. CHODL expression was silenced in the CACO2, CL14, DLD1, HCT116, HT29, LoVo, LS180, SW480, SW620 and SW1116 cell lines (Figure 1B). Therefore, our results demonstrated aberrant downregulation of CHODL in CRC.

### CHODL gene expression is epigenetically silenced in CRC

CHODL was transcriptionally silenced in the CRC cell lines but was readily expressed in normal human colon tissue. To investigate the mechanism that leads to the downregulation of CHODL in CRC, we searched for CpG islands in the promoter of CHODL (http://cpgislands.usc.edu/), and the region from -873 to 1577 relative to the transcription start site of CHODL was analyzed (Figure 1D). BGS revealed that the CRC cell lines, including DLD1, HCT116, HT29, LoVo, NCM and SW480, were hypermethylated in the promoter region of CHODL (Figure 1E). Moreover, demethylation treatment with 5-aza-2'-deoxycytidine in CACO2, DLD1, HCT116 and LS180 cells resulted in successful re-expression of CHODL, especially in DLD1 and HCT116(Figure 1F). To further substantiate the negative correlation between the level of promoter methylation in CHODL and its expression, we used data from Xena (Figure 1G). Taken together, these results showed that promoter methylation contributed to the aberrant silencing of CHODL in CRC cells.

### CHODL overexpression suppresses cell growth *in vitro*

**Figure 2:**
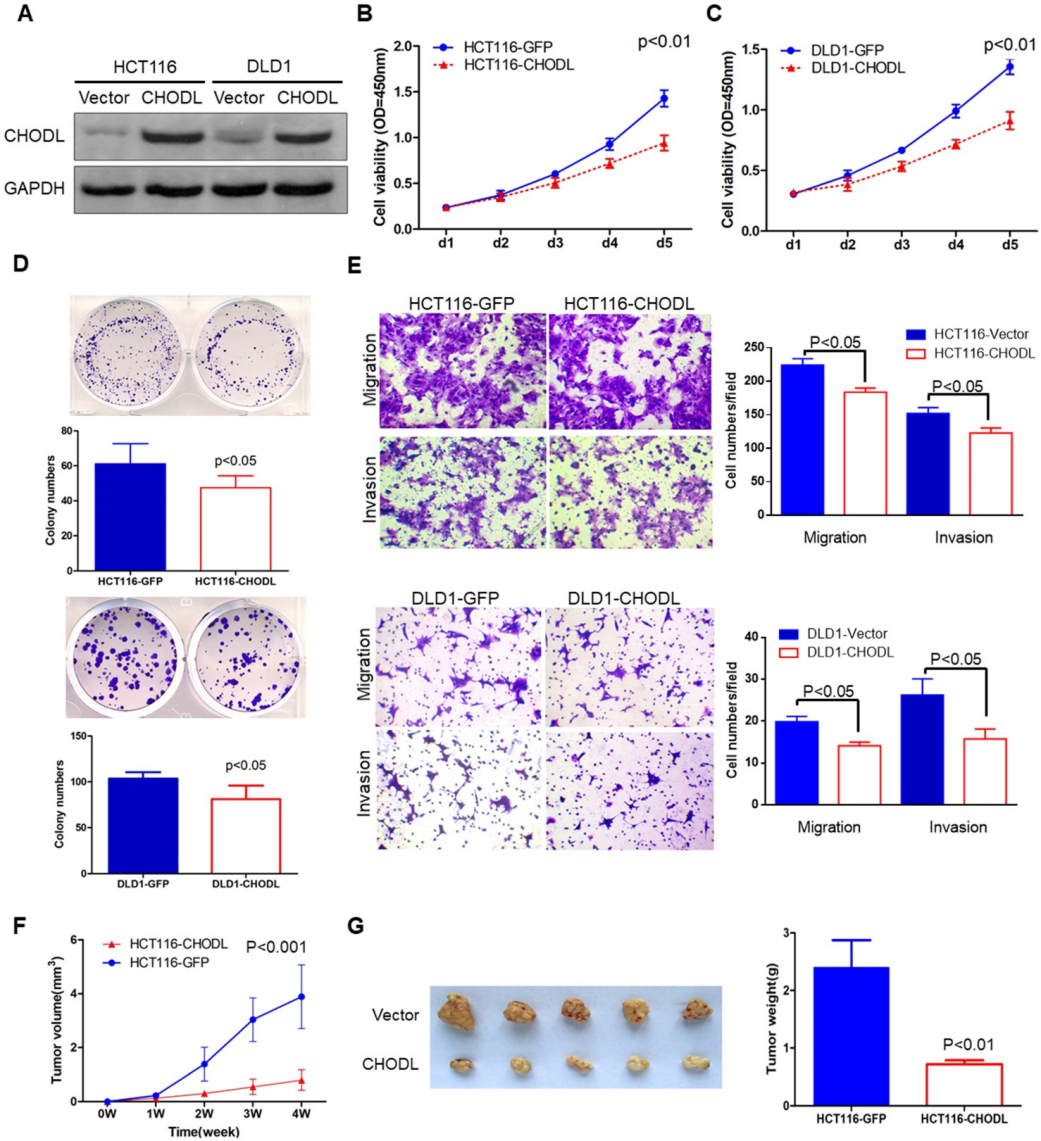
** CHODL inhibited malignant CRC phenotypes *in vitro* and *in vivo*.** (A) The ectopic expression efficiency of CHODL in HCT116 and DLD1 cells was confirmed by western blot analysis. (B. C) Cell viability was assessed by the MTS assay, which showed that the proliferative ability was lower in the CHODL-overexpressing cell lines than in the control cell lines. (D) The colony formation assay showed that the number of colonies was lower in the CHODL-overexpressing cell lines than in the control cell lines. (E) CHODL overexpression restrained cell migration and invasion in HCT116 and DLD1 cells. (F. G) Representative images of tumor growth in nude mice subcutaneously injected with HCT116 cells with control (GFP) and CHODL overexpression vectors. (F) CHODL overexpression suppressed tumor growth *in vivo*, and the tumor growth curves were plotted against days after treatment. (G) The weights of the tumors with or without CHODL expression are shown in a histogram and were compared by Student's t test.

The silencing of CHODL in CRC cell lines and tissues suggests that CHODL has a tumor-suppressive function in CRC tumorigenesis. To demonstrate the functional role of CHODL in CRC, we generated two stably transfected CRC cell lines (HCT116 and DLD1) with CHODL overexpression via lentivirus transfection. Ectopic expression of CHODL was confirmed by western blot analysis (Figure 2A). MTS [3-(4,5-dimethylthiazol-2-yl)-5-(3-carboxymethoxyphenyl)-2-(4-sulfophenyl)-2H-tetrazolium] cell viability assays and colony formation assays were used to examine the effect of CHODL on CRC cell growth. Ectopic expression of CHODL significantly inhibited cell viability (Figure 2B & C) and CRC colony formation (Figure 2D) compared with those of the control vector-transfected cells in both HCT116 and DLD1 cells. These results demonstrated the significant growth inhibitory effect of CHODL on CRC cell growth.

### CHODL reduces migration and invasion

Given the important role of lectins in the cytoplasm, cytoplasmic membrane and extracellular space, we investigated whether CHODL could alter cell migration and invasion. Transwell migration and Matrigel invasion assays were performed. Quantitative transwell analyses at 24 hours confirmed a significant decrease in the number of cells crossing the porous filter by chemotaxis after CHODL overexpression (18% in HCT116 cells and 19% in DLD1 cells, p<0.05, Figure 2E). A quantitative Matrigel invasion assay at 48 hours also indicated a reduced number of cells ectopically expressing CHODL that crossed the porous filter compared with that of the control cells (29% in HCT116 cells and 40% in DLD1 cells, p<0.05, Figure 2E). These results indicate that CHODL restrains CRC cell migration and suppresses the malignant phenotype of CRC.

### CHODL inhibits tumorigenicity in nude mice

To further explore the tumorigenic ability of CHODL *in vivo*, we injected empty vector-transfected [HCT116-green fluorescent protein (GFP)] and CHODL-transfected HCT116 (HCT116-CHODL) cells into the right flanks of BALB/c nude mice. The tumor growth rates of the nude mice injected with the HCT116-CHODL cells were significantly slower than those of the control group (Figure 2F). Twenty-eight days after injection, all mice were sacrificed. The average tumor weight of the nude mice injected with HCT116-CHODL cells (0.72±0.47 g) was significantly lower than that of the control mice (2.4±0.07 g, p<0.01, Figure 2G). Our results from the *in vivo* model provide additional support for the tumor-suppressive role of CHODL in CRC.

### Ectopic expression of CHODL promotes apoptosis

**Figure 3:**
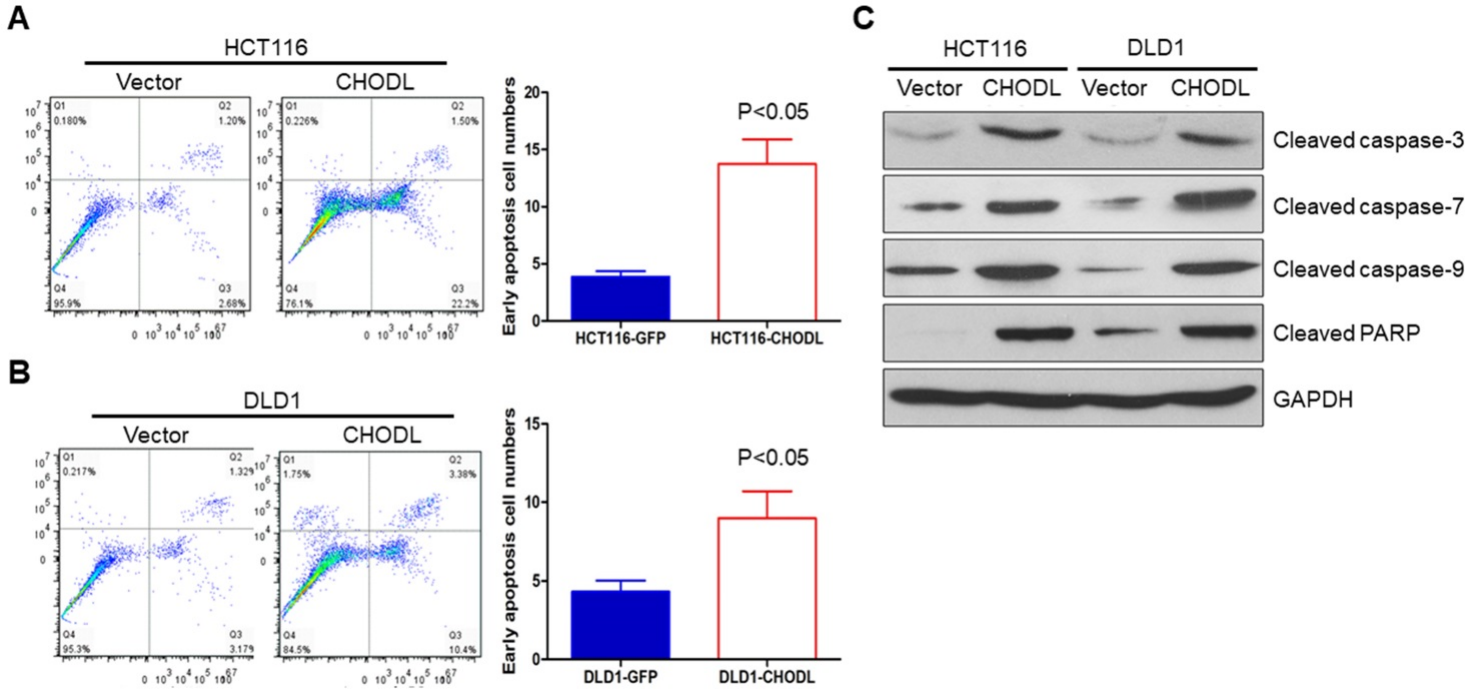
** CHODL induced apoptosis of CRC cells.** (A) The ectopic expression of CHODL promoted apoptosis, as shown by flow cytometry analysis of HCT116 cells stained with annexin V/7-AAD. (B) The ectopic expression of CHODL promoted apoptosis, as shown by flow cytometry analysis of DLD1 cells stained with annexin V/7-AAD. (C) CHODL induced protein expression of the active forms of caspase-3, caspase-7, caspase-9 and PARP, as shown by western blot analysis.

We further examined whether apoptosis contributes to the CHODL-mediated growth inhibition in CRC cells by performing annexin V-APC-fluorescence-activated cell sorting (FACS) apoptosis analyses. The results showed an obvious increase in the number of early apoptotic cells among the CHODL-overexpressing HCT116 cells compared with the control cells (3.88±0.28% vs 13.73±1.24%, p<0.05) (Figure 3A). The same effect was also observed in the CHODL-overexpressing DLD1 cells, where an increased proportion of early apoptotic phase cells was observed compared with that of the control cells (4.32±0.41% vs 8.97±0.99%, p<0.05, Figure 3B). Furthermore, we examined important regulators of apoptosis and found that CHODL significantly elevated the protein levels of the active forms of caspase-3, caspase-7, caspase-9 and poly ADP ribose polymerase (PARP) in both HCT116 and DLD1 cells (Figure 3C). Our results indicate that CHODL promotes cancer cell apoptosis in CRC.

### Overexpression of CHODL results in the deregulation of gene expression profiles and signaling pathways in CRC

**Figure 4:**
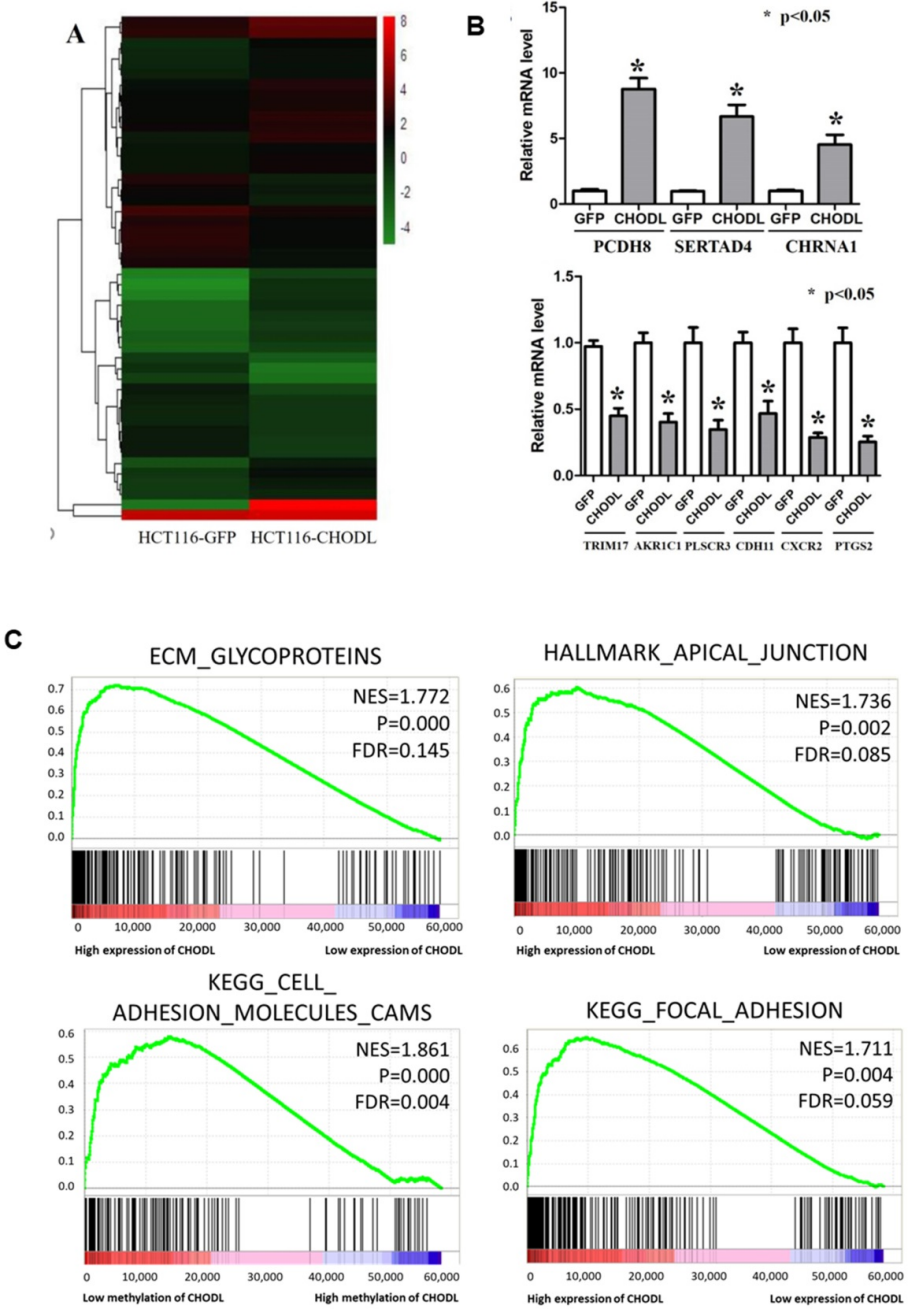
** Differentially expressed genes in CRC cells after the overexpression of CHODL.** (A) Heat map analysis of the number of differentially expressed genes identified in this study. Red indicates upregulated expression; green indicates downregulated expression. (B) qRT-PCR analysis of the expression of 9 genes selected from the RNA-Seq results. The y-axis shows the gene expression levels after normalization to the reference gene β-actin. (C) The differentially expressed genes were classified by GSEA.

To define the molecular mechanisms by which CHODL executes its tumor-suppressive function in CRC, we performed RNA sequencing analysis. In total, 1387 differentially expressed genes (fold-change >2, p<0.05) in CHODL-overexpressing cells were identified (Figure 4A); 891 were upregulated and 496 downregulated (Table S1). We validated several of these potential CHODL-related downstream genes, including PCDH8, SERTAD4, CHRNA1, TRIM17, AKR1C1, PLSCR3, CDH11, CXCR2 and PTGS2, in HCT116 cells by qRT-PCR analyses (Figure 4B). However, due to limitations of the RNA sequencing analysis and follow-up qRT-PCR analysis, we decided to examine more CRC tissues. Therefore, we ranked the RNA sequencing data from the UCSC Cancer Browser Database by the CHODL expression level in CRC tumor tissues and selected the top ten and bottom ten samples to perform a gene set enrichment analysis (GSEA). Our results showed that genes in the CHODL-overexpressing samples were closely associated with invasion-related pathways, including the extracellular matrix (ECM)- glycoprotein interaction, the apical junction pathway, focal adhesion (Figure 4C) and the ECM-receptor interaction. The invasive and metastatic abilities of cancer cells are enhanced when cell-cell or cell-ECM contacts are altered. Apical junctions, indicators of maturation of cell-cell contacts 14, 15, were increased in samples with high CHODL expression. The genes were also enriched in ECM glycoproteins, such as transmembrane molecule proteoglycans, a kind of glycoprotein that enhances interactions between cells and the ECM 16. Intriguingly, we found strong significant enrichment in the mTORC1 and MYC pathways (Supplementary Figure S1A, B). Having shown that CHODL overexpression is related to several important pathways, we then determined whether we could detect similar pathways in samples in which the CHODL promoter was hypomethylated. As expected, pathways related to cell adhesion phenotypes, apical junctions (Supplementary Figure S1C) and cell adhesion molecules (Figure 4C) were enriched. Collectively, these data indicate that the effects of CHODL inactivation on specific cellular pathways are important in cancer development.

### CHODL is a potential predictor for the survival of CRC patients

**Figure 5:**
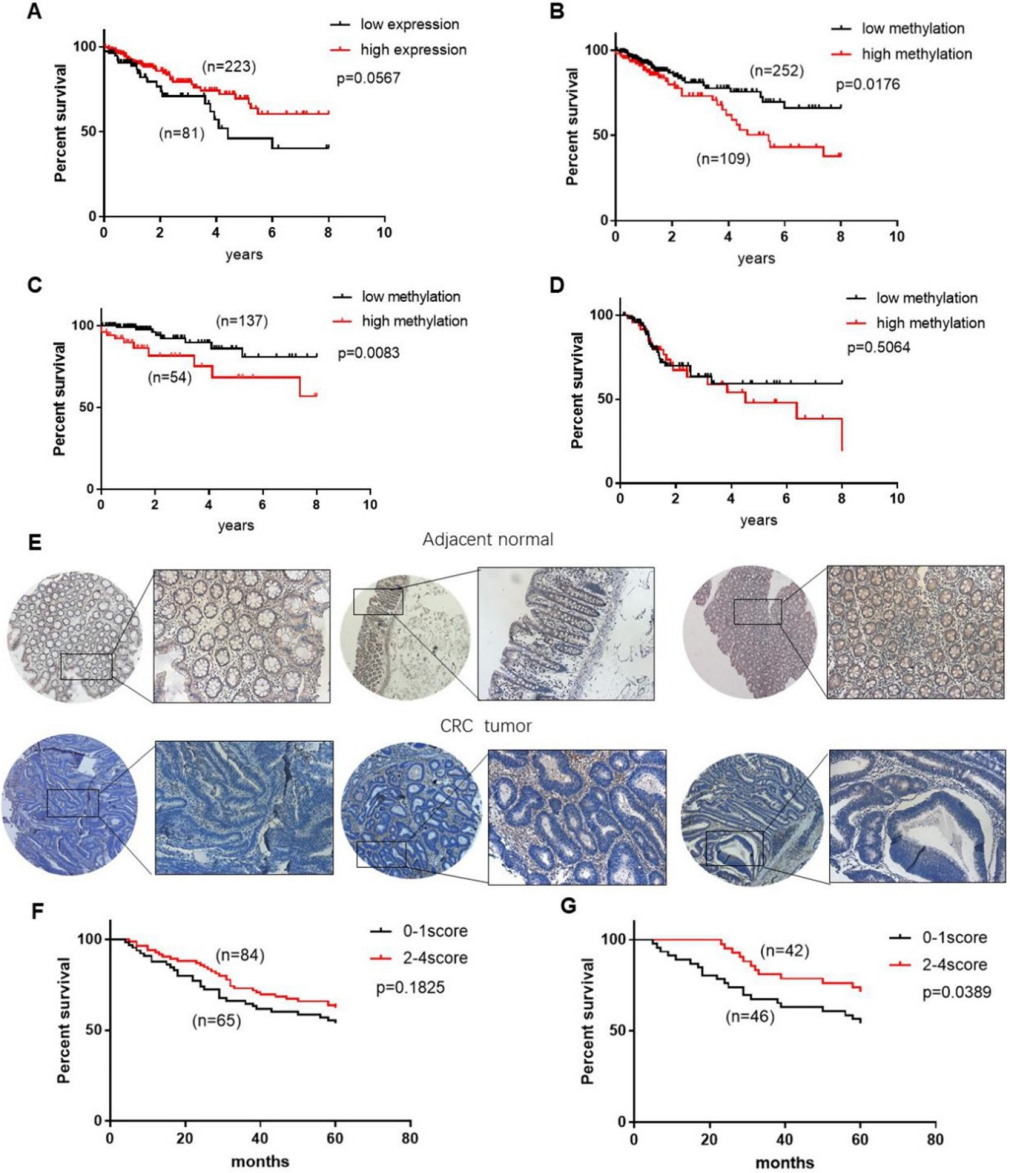
** CHODL may be a potential biomarker for the survival of CRC patients.** (A) Patients with high CHODL expression had better overall survival than those with low CHODL expression. (B) Patients with CHODL hypomethylation had better overall survival than those without hypomethylation. (C, D) The hypermethylation of CHODL may predict poor survival in early-stage patients but not in late-stage patients. (E) CHODL staining was decreased in CRC tumor tissues compared with normal tissues. (F, G) CHODL hyperexpression may predict better survival in CRC, especially in rectal cancer. Significance was determined using Kaplan-Meier analyses.

The above data showed that CHODL is silenced by promoter hypermethylation and functions as a tumor suppressor in CRC carcinogenesis. To examine the potential clinical role of CHODL expression and methylation status in CRC, we analyzed the CHODL mRNA expression and methylation data of CRC patients combined with their clinical information from TCGA. As shown by the Kaplan-Meier survival curve (Figure 5A), CRC patients with low CHODL mRNA expression (n=81) showed poorer survival than patients with high CHODL mRNA expression (n=223), although the difference was not significant (p=0.056). Accordingly, we examined whether the methylation level of CHODL could predict patient survival. As expected, when the methylation level of the CRC samples was dichotomized based on a cut-off score value of 0.55538, the overall survival associated with CRC samples with a relatively low methylation status (n=252) was significantly better than that of samples with a high methylation status (n=109, p<0.05 log-rank test, Figure 5B), especially in the early stage (p<0.05 log-rank test, Figure 5C, D). The associations between clinical features and CHODL methylation in CRC are shown in Table 1. There was no correlation between the methylation of CHODL and pathologic stage, age, or race, but there was a correlation with sex (p=0.022). There were no significant differences in age, sex or race by vital status (Table 2), but obvious differences in CHODL methylation and tumor-node-metastasis (TNM) stage were observed. In the univariate Cox regression analysis (Table 3), tumor stage and patient age were significant prognostic factors (p<0.0001), and the methylation level of CHODL was also associated with an increased risk of cancer-related death (relative risk [RR], 0.467; 95% confidence interval [CI], 0.269-0.809; p<0.05). After adjustment for potential confounding factors, the multivariate Cox regression analysis demonstrated that the CHODL methylation level was an independent predictor of poor survival of CRC patients (RR, 0.508, 95% CI, 0.281-0.921, p<0.05) (Table 4). Moreover, we performed IHC staining to determine the level of CHODL in CRC. Based on the staining percentage, we grouped the CRC samples according to CHODL expression with a score of 0 to 4 (Figure S2). CHODL staining was decreased in CRC tumors compared with normal tissues (Figure 5E). Analysis of the clinical data for IHC samples showed that high CHODL expression in rectal cancer samples (p<0.05), but not all CRC samples, was associated with a significantly improved prognosis (Figure 5F, G). These results suggest that CHODL hypermethylation is a predictor of poor survival in CRC patients and that the expression of CHODL may predict improved prognosis in rectal cancer.

## Discussion

The aberrant hypermethylation of CpG islands is an important hallmark of CRC that can lead to the aberrant silencing of tumor suppressor genes and cancer formation, and the CIMP has been shown to be an independent negative prognostic factor in CRC patients 17. To identify novel tumor suppressor genes regulated by the CIMP, we used methyl-DNA immunoprecipitation assays to identify a novel methylated gene, CHODL, which is preferentially methylated in human CRC 18.

CHODL belongs to C-type lectin-containing protein family which are involved in diverse processes including cell recognition and communication, cell-cell adhesion, and extracellular matrix (ECM)-cell interactions 9. In the motor neuron degenerative disease spinal muscular atrophy model, CHODL was found to have mRNA splicing disorder which can affect motor neuron mature^11^,^19^. Masuda et al. found CHODL is important in lung carcinogenesis and metastasis and may be a potential diagnostic and prognostic marker for lung cancer. Other researches showed that overexpression of human CHODL was associated with the metastasis of hepatocellular carcinoma and was also showed that CHODL could be an independent prognostic factor in HCC based on TCGA and GEO database^13^,^20^.

In this study, we showed that CHODL was silenced in CRC cell lines and tumor tissues and frequently expressed in normal colonic tissues. BGS analysis demonstrated that the CHODL promoter in CRC cell lines was fully methylated and that the expression of CHODL was restored by demethylation treatment. Further verification using TCGA data showed that promoter hypermethylation of CHODL was the predominant mechanism for the silencing of the CHODL gene in CRC and that CHODL had a tumor-suppressive role in CRC.

Consistent with this hypothesis, we found that CHODL suppresses CRC cell proliferation, induces apoptosis, and inhibits cell migration and invasion. Our functional experiments showed that re-expressing CHODL in CRC cell lines inhibited cell growth and tumorigenicity both *in vitro* and *in vivo*. Next, we focused on the underlying mechanism by which CHODL inhibits cell growth. Apoptosis analysis showed that the re-expression of CHODL in CRC cell lines induced apoptosis and led to an increased proportion of cells in early apoptosis. Western blot analysis confirmed that apoptosis was mediated by the caspase-dependent apoptosis pathway, which involves caspase-9, caspase-3, caspase-7 and PARP 21, 22. RNA-Seq analysis further showed that CHODL regulates apoptosis in CRC. TRIM17 and PLSCR3 were downregulated by CHODL. TRIM17 acts as an inhibitor of bulk autophagy by forming complexes with Beclin-1 23, and the antiapoptotic protein Mcl-1 functions as an E3 ubiquitin ligase 24. Phospholipid scramblase 3 (PLSCR3) can be phosphorylated by protein kinase C-delta (PKC-delta) and plays pivotal roles in regulating the apoptotic response 25.

Additional functional experiments demonstrated that CHODL could restrain cell invasion and metastasis. This finding indicates that CHODL exerts its antitumor effect on the ECM and the epithelial- mesenchymal transition (EMT) process, and relevant evidence was found by GSEA. The human CHODL protein belongs to the C-type lectin-containing protein family, which has been reported to play a significant role in the structure and stability of the ECM and the interactions between the ECM and cells 26-27. Previous studies have suggested that CHODL suppresses tumor migration and invasion via EMT 28. Our RNA-Seq data suggested that some EMT-related genes (AKR1C1, CDH11, PCDH8, and CXCR2) were differentially expressed due to CHODL in CRC. AKR1C1, a member of the human aldo-keto reductase family, was downregulated according to our RNA-Seq analysis, and it exerts its prometastatic effects by directly interacting with STAT3, facilitating its phosphorylation and reinforcing STAT3 binding to the promoter regions of JAK2, which might promote metastasis in a catalytic-independent manner 29. CDH11 is a mesenchymal cadherin that promotes cell migration by enhancing TGFβ-stimulated signaling 30. Lee YC et al. reported that targeting the extracellular domain of CDH11 could restrict cellular adhesion and metastatic dissemination in cancer cells 31. PCDH8 is located within a cluster of protocadherins conserved between humans and mice and inhibits the proliferation and migration of cancer cells by expanding epithelial cells 32. CXCR2, a receptor of chemokines and IL-8, is believed to be responsible for endothelial cell chemotaxis and regulates cell migration to sites of inflammation 33,34. This molecule may be involved in tumorigenesis and the metastatic process by recruiting myeloid cells in the tumor microenvironment and the metastatic niche 35. Through functional assays and RNA-Seq, we identified a relationship between low CHODL expression and malignant cancer phenotypes, but the mechanism by which CHODL suppresses malignancy in CRC is still unclear. mTORC1 is a molecule of interest. As a nutrient, energy sensor and mediator of protein synthesis, mTORC1 exerts its powerful functions by facilitating the proliferation, survival, invasion and metastasis of tumor cells 36. By utilizing GSEA, we found that genes modulated by mTORC1 activation are significantly correlated with low CHODL expression. This result led us to hypothesize that CHODL may be an inhibitor of mTORC1. Another possible mechanism by which CHODL suppresses malignancy is through the MYC-related pathway, which, according to GSEA, is associated with low CHODL expression. MYC, a well-known oncogene, was shown to drive cell proliferation and regulate cell growth, apoptosis, differentiation, and stem cell self-renewal 37. CHODL may suppress tumor malignancy in an anti-MYC manner. The mechanism of CHODL in CRC needs to be elucidated to confirm this hypothesis.

Based on the tumor-suppressive role of CHODL identified both *in vitro* and *in vivo*, we further investigated the clinical value of CHODL as a predictor of CRC prognosis. We analyzed TCGA database, which contains 597 CRC cases with complete follow-up data, to examine CHODL mRNA expression and methylation levels. The results were consistent with our *in vitro* data, showing that CHODL was downregulated in CRC tissues compared with adjacent normal tissues. We discovered that low methylation of CHODL in CRC was an independent predictor of improved survival. All these findings showed that CHODL mRNA expression and methylation level could be a molecular signature in the development of CRC. Collectively, the findings of this research are consistent with the hypothesis that the aberrant epigenetic inactivation of CHODL contributes to CRC tumorigenesis and that CHODL acts as a tumor suppressor in CRC development. A low CHODL methylation level may serve as an independent biomarker to predict improved survival for CRC patients. Nevertheless, the network involved must be determined to help define the mechanism of CHODL.

## Materials and Methods

### Clinical samples

Twenty-four paired biopsy specimens from the primary tumor and adjacent nontumor sites from patients with CRC were collected from the First Affiliated Hospital, Sun Yat-sen University, Guangzhou, China. The study protocol was approved by the Clinical Research Ethics Committee of the First Affiliated Hospital, Sun Yat-sen University, Guangzhou, China.

### Cell culture

The human colon cancer cell line HCT116 was grown in McCoy's 5A medium (Invitrogen), whereas the CACO2, CL14, DLD1, HT29, LoVo, LS180, NCM, SW480, SW620 and SW1116 cell lines were grown in Roswell Park Memorial Institute (RPMI) 1640 medium (Invitrogen). All media were supplemented with 10% fetal bovine serum (FBS, HyClone).

### Transfection and overexpression of CHODL

Full-length CHODL was amplified by PCR and cloned into the lentiviral expression vector pLVX- IRES-neo (Clontech Laboratories, San Francisco, CA, USA) according to the manufacturer's instructions. CRC cells at 50% confluence in 6-well plates were transfected with the lentivirus using Lipofectamine 2000 (Invitrogen) at room temperature. RNA and proteins were harvested at 48 or 72 hours after transfection. Puromycin was used to select stably transfected cells.

### Demethylating treatment with 5-aza-dC

CRC cells were cultured in 60 mm dishes and treated with 10 μM 5-aza-dC (Selleck, USA, S1200). After 72 hours of treatment, the cells were harvested for subsequent analysis.

### DNA extraction and bisulfite conversion

Genomic DNA from CRC cell lines was isolated using a TIAN-amp Genomic DNA Kit (TIANGEN Biotech, Beijing, DP304). Two micrograms of DNA was modified by sodium bisulfite using an EZ DNA Methylation-Gold Kit (Zymo, USA, D5006).

### Methylation-specific PCR and BGS

Methylation-specific PCR was used to amplify the bisulfite-modified genomic DNA, and primers were designed to identify the methylation and nonmethylation sites. The PCR products were sequenced and analyzed by FinchTV. The primers used throughout this experiment were as follows: MSP-F: 5'-GTGCGTTTTGGGTAGAGGTC-3', MSP-R: 5'-CCCAACAACAACGAAACCACG-3'; USP-F: 5'-GTTGTGTTTTGGGTAGAGGTTG-3', USP-R: 5'-ACCCAACAACAACAAAACCACA-3'; and BGS-F: 5'-AGGGAGGTTGTAGAGTTAGAGT-3', BGS-R: 5'-CCTTACACCATACAAATTTCCAA-3'.

### RNA extraction, semiquantitative RT-PCR and real-time PCR analyses

QIAzol reagent (Qiagen) was used to extract total RNA from CRC cell pellets or tissues. cDNA was synthesized using a GoScript Reverse Transcription Kit (Promega, USA, A5001). PCR was performed to detect relative CHODL expression. Semiquantitative RT-PCR was performed using Hot Start DNA Polymerase (Invitrogen). The housekeeping gene β-actin was used as an internal control. Real-time PCR was carried out using SYBR Green master mix on an HT7900 system according to the manufacturer's instructions (Applied Biosystems). The primers used in this experiment were as follows: CHODL-RT-sense: 5'-GGAAGGAAAGGAACTACGAAATC-3', CHODL- RT-antisense: 5'-GTTAAAAGGAGCACAGGGACAT A-3'.

### Western blot analysis

Total protein was extracted. The protein concentration was measured by the DC protein assay method of Bradford (Bio-Rad). Protein samples (30 µg) were separated on a Bis/Tris-polyacrylamide gel by electrophoresis and then blotted onto nitrocellulose membranes (GE Healthcare). Blots were incubated with the primary antibodies overnight at 4°C and with the secondary antibody for 1 hour at room temperature. ECL Plus Western Blotting Detection Reagents (GE Healthcare) were used to visualize the proteins.

### Cell viability assay

The MTS assay was performed to assess cell viability according to the manufacturer's instructions (Promega, Madison, WI). Briefly, 3×103 cells were seeded into a 96-well plate, and 100 µl of culture medium and 20 µl of reaction solution containing 333 µg/ml MTS and 25 µM phenazine ethosulfate were added to the cells. The mixture was incubated at 37°C for 1 hour, and the optical density (OD) was measured at 450 nm.

### Colony formation assay

Stable CHODL-overexpressing and control cells were collected and seeded (2×103/well) into 6-well plates for 14 days. Colonies (50 cells/colony) were then counted after fixation with 70% ethanol and staining with crystal violet solution.

### Flow cytometry

For apoptosis analysis, CHODL-overexpressing and control cells were seeded into 6-well plates at a density of 5×10^5^ cells per well. Cells were harvested overnight at 37°C. Then, the cells were trypsinized, washed with phosphate-buffered saline (PBS), stained with annexin V (FITC-conjugated) (BD Biosciences) and 7-amino-actinomycin (7-AAD) (BD Biosciences) for 20 min, and analyzed immediately by FACS using a FACScan system (BD Biosciences). Cell populations were classified as viable (annexin V-negative, 7-AAD-negative), early apoptotic (annexin V-positive, 7-AAD-negative), late apoptotic (annexin V-positive, 7-AAD-positive), or necrotic (annexin V-negative, 7-AAD-positive).

### Transwell migration and invasion assays

A 24-well plate containing 8 mm pore size chamber inserts (Corning, USA) was utilized to evaluate the migration and invasion of CRC cells. For the migration assay, 1×105 CRC cells were positioned in the upper chamber. For the invasion assay, the membrane was coated with Matrigel (BD Biosciences, USA) to form a matrix barrier, and 2×10^5^ CRC cells were seeded into the upper chamber. In each lower chamber, 600 ml of Dulbecco's modified Eagle's medium (DMEM) supplemented with 10% FBS was added. CRC cells were incubated at 37°C and allowed to migrate for 36 hours or invade for 48 hours. After incubation, the CRC cells that had migrated or invaded through the pore were fixed with 4% paraformaldehyde and stained with 0.1% crystal violet. Then, the CRC cells were counted and photographed under an IX71 inverted microscope (Olympus, Tokyo, Japan). Five random fields were counted using an optical microscope.

### *In vivo* tumorigenicity

HCT116 cells (1.0×10^6^ cells in 100 µl of PBS) overexpressing CHODL or the control vector were injected subcutaneously into the dorsal right flanks of 4-week-old male BALB/c nude mice. Tumor diameter was monitored every 3 days for 3 weeks. Tumor volume (mm3) was calculated by measuring the longest and shortest diameter of the tumor. All experimental procedures were approved by the Animal Ethics Committee of the First Affiliated Hospital, Sun Yat-sen University, Guangzhou, China.

### RNA-Seq

After isolation, RNA was quantified (Qubit RNA Assay Kit, Life Technologies, Inc.), and RNA quality was assessed (RNA6000 Nano Kit and Bioanalyzer 2100, Agilent). RNA (1000 ng) was used as input for the VAHTSTM mRNA-seq v2 Library Prep Kit for Illumina® (Vazyme, Inc.). The sequencing libraries were established according to the manufacturer's protocol. Briefly, poly(A) RNA was purified via two rounds of hybridization to Dynal oligo(dT) beads. Poly(A)+ RNA was fragmented and then used for first- and second-strand cDNA synthesis with random hexamer primers. The cDNA fragments were treated with an End-It DNA End Repair Kit to repair the ends, modified with Klenow fragment to add an A at the 3' end of the DNA fragments, and finally ligated to the adapters. Ligated cDNA products were subjected to PCR amplification. The quality of the library product was established with a Bioanalyzer 2100 (Agilent). Then, the RNA-Seq libraries were sequenced using the Illumina HiSeq 4000 platform. Reads were matched to the human reference genome GRCh37/ hg19 using TopHat v2.1.0 (Langmead, 2012). The reads per kilobase of exon model per million mapped reads (RPKM) of each gene were calculated based on the length of the gene and the read counts mapped to the gene. Differential expression analysis of two conditions/groups (two biological replicates per condition) was performed using the previously described statistical model 21. The resulting p values were fitted by Benjamini and Hochberg's approach to determine the false discovery rate. Genes with an adjusted p value <0.05 were considered differentially expressed. KOBAS software was used to test the statistical enrichment of the differentially expressed genes in Gene Ontology (GO) and Kyoto Encyclopedia of Genes and Genomes (KEGG) pathways.

### Databases

CHODL mRNA expression data were downloaded from the UCSC Cancer Browser Database (https://genome-cancer.ucsc.edu/proj/site/hgHeatmap/). CHODL methylation data (lihc_tcga.tar; data_methylation_hm450) were downloaded from the Xena database (https://xena.ucsc.edu/). Survival data were extracted from TCGA. A total of 362 samples were analyzed in this study. A GSEA was applied to determine the biological pathway divergences between high and low CHODL expression.

### Statistical analysis

The results are expressed as the mean±standard deviation (SD). Statistical analysis and plotting were performed using the SPSS statistical software package (standard version 13.0, IBM, USA) and GraphPad Prism 6.0 (GraphPad Software, USA). The Mann-Whitney U test or Student's t test was performed to compare the pathologic variables of the two sample groups in the functional assay. The difference in cell viability and the tumor growth rate of nude mice between the two groups was determined by repeated-measures analysis of variance. A value of p<0.05 was regarded as statistically significant.

## Supplementary Material

Supplementary figures and tables.

## Figures and Tables

**Table 1:** Distribution of patient characteristics by the level of methylation of CHODL

		Low methylation		High methylation		p value
		n	%	n	%	
**TMN stage**	Early stage	138	72.3	53	27.7	0.384
	Late stage	115	68.0	54	32.0	
**Mean age, y±SD**		64±13		65±12		0.199
**Gender**	Male	126	64.9	68	35.1	0.022
	Female	127	76.0	40	24.0	
**Race**	White	188	69.4	83	30.6	0.270
	Asian	6	50.5	6	50.5	
	Black or African American	42	73.7	15	26.3	

**Table 2:** Distribution of patient characteristics by survival status

		Alive		Dead		p value
		n	%	n	%	
**TMN stage**	Early stage	172	90.0	19	10.0	<0.0001
	Late stage	123	72.7	46	27.3	
**Mean age, y±SD**		63±12.6		69±13.7		0.688
**Gender**	Male	155	79.9	39	20.1	0.335
	Female	140	83.8	27	16.2	
**Race**	White	219	80.8	52	19.2	0.625
	Asian	11	91.7	1	8.3	
	Black or African American	47	82.5	10	17.5	
**CHODL methylation**	Low methylation	216	85.4	37	14.6	0.006
	High methylation	79	73.1	29	26.9	

**Table 3:** Univariate Cox Regression Analysis of Potential Poor Prognostic Factors for CRC Patients

Variable		RR (95% confidence interval)	p value
**TMN stage**	Low stage	0.300 (0.175-0.513)	<0.0001
	High stage	1.00	
**Age**		1.031 (1.009-1.054)	0.007
**Gender**	Male	1.244 (0.761-2.034)	0.383
	Female	1.00	
**Race**	White	0.914 (0.463-1.804)	0.796
	Asian	0.780 (0.099-6.115)	0.813
	Black or African American	1.00	
**CHODL** **methylation**	Low methylation	0.619 (0.308-1.000)	0.05
	High methylation	1.00	

**Table 4:** Multivariate Cox Regression Analysis of Potential Poor Prognostic Factors for CRC Patients

		Model A			Model B	
Variable		RR (95% CI)	p value		RR (95% CI)	p value
**TNM stage**	Early stage	0.299 (0.166-0.537)	<0.0001		0.243 (0.132-0.447)	<0.0001
	Late stage	1.00				
**CHODL Methylation**	Low methylation	0.493 (0.279-0.873)	0.025		0.508(0.281-0.921)	0.026
	High methylation	1.00			1.00	
**Age**					1.047 (1.022-1.073)	<0.0001
**Gender**	Male				1.097 (0.612-1.965)	0.757
	Female				1.00	

Model A: exclude age and gender; Model B: include age and gender

## Abbreviations

CRC: Colorectal cancer
CIMP: CpG island methylated phenotype
TCGA: The Cancer Genome Atlas
GSEA: Gene set enrichment analyses
BGS: Bisulfite genomic sequencing
