# Supplementary material for: Promoter Hypermethylation of CHODL Contributes to Carcinogenesis and Indicates Poor Survival in Patients with Early-stage Colorectal Cancer
Source: J Cancer. 2020 Feb 28;11(10):2874–86. doi: 10.7150/jca.38815 (PMC7086254; doi:10.7150/jca.38815)
Supplement: Supplementary file 1 — Supplementary figures and tables. [file jcav11p2874s1.pdf]

Supplementary Materials

Table S1

Part of differentially expressed genes in CHODL over-expressed cells

Gene Up-regulated	Fold-change (p<0.05)	Gene Down-regulated	Fold-change (p<0.05)
LOC100129203	23	TRIM17	11
RAET1E-AS1	17	AKR1C1	10
HOXD-AS2	15	PLSCR3	10
CHRNA1	13	CDH11	9
RPRML	13	TULP2	8
SERTAD4	13	CXCR2	8
PRR34	13	PTGS2	7
ANGPTL6	13		
LOC100506747	13		
PCDH8	13		

Figure S1

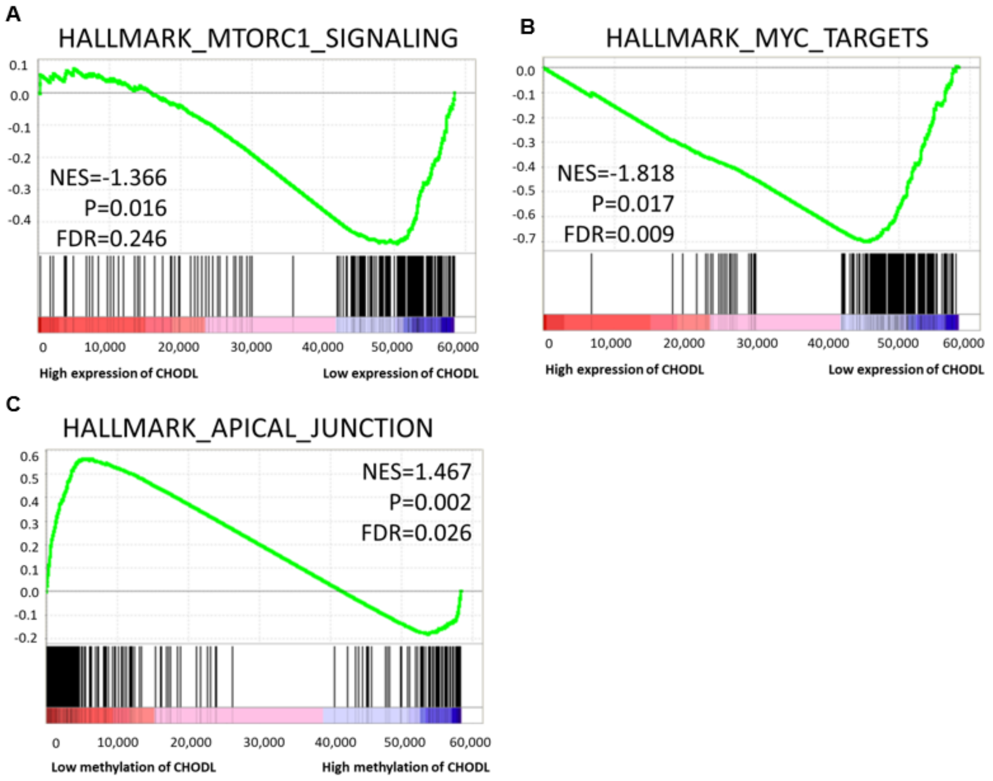

Figure S1 Classified the differentially expressed genes by GSEA.

Figure S2

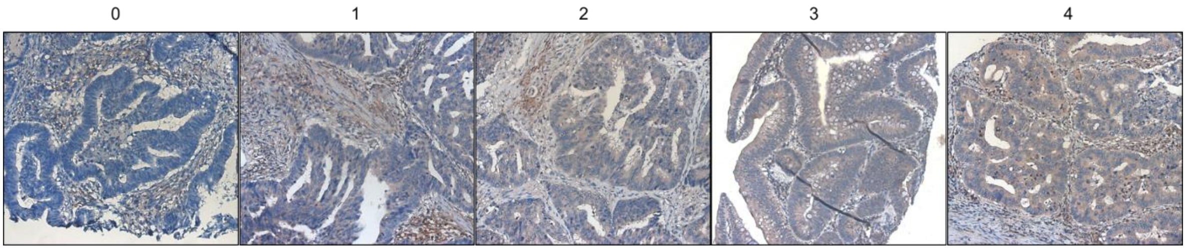

Figure S2 Scores indicated CHODL levels in representative tumor tissues as determined by IHC staining. Scores were calculated by intensity and percentage of stained cells. Scale bars, 100 um.
